# Obesity aggravates acute kidney injury resulting from ischemia and reperfusion in mice

**DOI:** 10.1038/s41598-024-60365-3

**Published:** 2024-04-29

**Authors:** Igor Oliveira da Silva, Nicole K. de Menezes, Heloisa D. Jacobina, Antonio Carlos Parra, Felipe Lima Souza, Leticia Cardoso Castro, Joris J. T. H. Roelofs, Alessandra Tammaro, Samirah Abreu Gomes, Talita Rojas Sanches, Lucia Andrade

**Affiliations:** 1 Laboratory of Basic Science in Renal Diseases (LIM-12), Division of Nephrology, University of São Paulo School of Medicine, São Paulo, Brazil; 2 Laboratory of Cellular Genetic and Molecular Nephrology, Division of Nephrology, Av. Dr. Arnaldo, 455, 3º Andar, sala 3310, University of São Paulo School of Medicine, São Paulo, SP CEP 01246-903 Brazil; 3 Department of Pathology, Amsterdam UMC, Location AMC, University of Amsterdam, Amsterdam, the Netherlands; 4 Amsterdam Cardiovascular Sciences, University of Amsterdam, Amsterdam, the Netherlands

**Keywords:** Kidney, Fat metabolism

## Abstract

In critically ill patients, overweight and obesity are associated with acute respiratory distress syndrome and acute kidney injury (AKI). However, the effect of obesity on ischemia–reperfusion injury (IRI)-induced AKI is unknown. We hypothesized that obesity would aggravate renal IRI in mice. We fed mice a standard or high-fat diet for eight weeks. The mice were divided into four groups and submitted to sham surgery or IRI: obese, normal, normal + IRI, obese, and obese + IRI. All studies were performed 48 h after the procedures. Serum glucose, cholesterol, and creatinine clearance did not differ among the groups. Survival and urinary osmolality were lower in the obese + IRI group than in the normal + IRI group, whereas urinary neutrophil gelatinase-associated lipocalin levels, tubular injury scores, and caspase 3 expression were higher. Proliferating cell nuclear antigen expression was highest in the obese + IRI group, as were the levels of oxidative stress (urinary levels of thiobarbituric acid-reactive substances and renal heme oxygenase-1 protein expression), whereas renal Klotho protein expression was lowest in that group. Expression of glutathione peroxidase 4 and peroxiredoxin 6, proteins that induce lipid peroxidation, a hallmark of ferroptosis, was lower in the obese + IRI group. Notably, among the mice not induced to AKI, macrophage infiltration was greater in the obese group. In conclusion, greater oxidative stress and ferroptosis might aggravate IRI in obese individuals, and Klotho could be a therapeutic target in those with AKI.

## Introduction

Obesity is often a chronic, progressive disorder, leading to poor health and an increased risk of death. In 2017, a high body mass index accounted for 4.72 million deaths and 148 million disability-adjusted life-years worldwide and was the fourth leading risk factor for mortality^1^. Obesity and oxidative stress are deeply interconnected. The increase in the quantity of adipose tissue leads to ectopic fat deposition in many organs, including the kidneys^2^. The increased production of reactive oxidative species (ROS) and oxidative stress in adipose tissue can have various pathological manifestations^3^. It has also been demonstrated that an increase in visceral adiposity is associated with a decrease in serum Klotho protein levels; therefore, obesity induces a state of Klotho deficiency^4^. Obesity has been associated with acute kidney injury (AKI) in intensive care unit patients and in patients recovering from surgery^5–8^. In one systematic review and meta-analysis, the risk of AKI was found to be higher among patients who were overweight or obese than among those who had a normal body mass index^9^. Ribeiro et al.^10^ demonstrated that cisplatin-induced AKI was exacerbated in obese mice, also showing that the degree of renal oxidative stress was higher in those mice. In addition, obesity is now considered an independent risk factor for chronic kidney disease^11,12^. During the coronavirus disease 2019 pandemic, obesity was found to be a risk factor for AKI and for AKI-related mortality^13–16^.

Despite the fact that obesity is a known risk factor for AKI, there have, to our knowledge, been no animal studies demonstrating the role that obesity plays in renal ischemia–reperfusion injury (IRI)-induced AKI. We hypothesized that obesity would aggravate renal IRI in a Klotho/ROS-dependent pathway in mice.

## Methods

### Experimental design

All experimental procedures were approved by the Medical and Research Ethics Committee of the University of São Paulo School of Medicine (Animal Ethics Committee, No. 1177/2018) and were conducted in accordance with the National Institutes of Health Guide for the Care and Use of Laboratory Animals. The study is reported in accordance with the ARRIVE guidelines. Six-week-old male C57BL/6 mice were purchased from the animal facility of the University of São Paulo School of Medicine. The mice were randomly divided into two groups and fed one of two diets over an eight-week period: a high-fat diet (RH19532: protein 20.1%, carbohydrates 44.3%, fat 18.9%, sodium 0.16%, potassium 0.38%, kcal/g 4.3; Rhoster, Araçoiaba da Serra, Brazil); or a standard diet (RH19521: protein 13.45%, carbohydrates 68.9%, fat 3.8%, sodium 0.14%, potassium 0.30%, kcal/g 3.6; Rhoster). The mice were randomly divided into four groups. Those in two of the groups were fed a standard diet for eight weeks, after which they were submitted to sham surgery (normal; n = 6), or renal artery clamping for 30 min (normal + IRI; n = 12). Those in the other two groups were fed a high-fat diet for eight weeks, thereafter being submitted to sham surgery (obese; n = 6), or renal artery clamping for 30 min (obese + IRI; n = 12). All studies were performed 48 h after sham surgery or IRI.

### Metabolic cage studies

At baseline, at week 4, and at 24 h after sham surgery or IRI, the mice were moved to individual cages and maintained on a 12:12-h light–dark cycle with ad libitum access to water only (no food provided), to collect 24-h urine samples^16^. At 48 h after IRI surgery, water intake was quantified and urine volume was measured. Animals were then anesthetized with intraperitoneal injections of ketamine (90 mg/kg body weight) and xylazine (10 mg/kg body weight), after which blood samples were collected. The kidneys were removed immediately thereafter. Some kidneys were frozen in liquid nitrogen and stored at − 70 °C for subsequent immunoblotting. Renal tissues were also processed for immunohistochemistry and histological analyses. Biochemical analyses were performed in plasma and urine.

### Mouse model of IRI

Prior to IRI induction, mice were anesthetized with ketamine (90 mg/kg body weight) and xylazine (10 mg/kg body weight). The kidneys were exposed through a midline incision, after which both renal arteries were clamped for 30 min and released. The entire procedure was performed on a heated bed. The mice in the normal and obese groups were submitted to the same procedure without clamping of the arteries. At the end of the procedures, the mice were left to rest in isolated cages. The cages were kept on top of a heated blanket to maintain constant body temperatures. The mice were kept under surveillance until they showed signs of activity, at which point morphine (10 mg/kg BW) was injected into the subcutaneous region behind the head to reduce postoperative pain and speed recovery. During the recovery period, we also added dipyrone to the drinking water (200 mg/kg BW). At 24 h after the surgical procedures, the mice were moved to metabolic cages, where they remained for an additional 24 h. All studies were performed 48 h after the surgical procedures.

### Analysis of blood and urine

Urine and blood samples were centrifuged in aliquots for 30 min at 4,000 g. Serum and urinary levels of sodium and potassium were measured with an auto-analyzer (EasyLyte; Medica Corporation, Bedford, MA, USA). Creatinine was measured with a commercial kit (Creatinine kit; Labtest Diagnóstica, Lagoa Santa, Brazil). Creatinine clearance was calculated with the following formula:

$$Creatinine\;clearance = \left[ {U_{creat} \times \left( {U_{volume} /T} \right)} \right]{ /}P_{creat}$$

where *U*_*creat*_ is the urinary concentration of creatinine (in mg/dL), *U*_*volume*_ is the urine volume (in µL), *T* is the time (in min), and *P*_*creat*_ is the plasma concentration of creatinine (in mg/dL).

### Assessment of reactive oxygen metabolites

Urinary levels of thiobarbituric acid reactive substances (TBARS) were assessed (in nmol/mL) with a commercial kit (TBARS Assay Kit; Cayman Chemicals, Ann Arbor, MI, USA). The detection system and quantification followed the protocols provided by the manufacturer. Absorbance was determined with a microplate spectrophotometer (Epoch 2; BioTek Instruments, Winooski, VT, USA).

### Measurement of urinary neutrophil gelatinase-associated lipocalin

Urinary neutrophil gelatinase-associated lipocalin (NGAL) was measured by using a commercially available ELISA kit (R&D Systems, Minneapolis, MN, USA).

### Western blot analysis

#### Kidney fractions

Kidney samples were homogenized as previously described^17^. Homogenates were centrifuged at 4000×*g* for 30 min at 4 °C, to remove nuclei and cell debris. Protein in the supernatant was quantified by bicinchoninic acid assay (Pierce BCA Protein Assay Kit no. 23225; Thermo Fisher Scientific, Waltham, MA, USA).

#### Electrophoresis

As previously described^18^, kidney samples were run on polyacrylamide minigels. After transfer by electroelution to polyvinylidene difluoride membranes (GE Healthcare, Little Chalfont, UK), blots were blocked with 5% nonfat dry milk in Tris-buffered saline. Blots were then incubated overnight with antibodies against caspase 3 (1:500), heme oxygenase-1 (HO-1, 1:1,000), peroxiredoxin 6 (Prdx6, 1:1,000), and nitrotyrosine (1:1,000), all of which were obtained from Abcam (Cambridge, UK); and against glutathione peroxidase 4 (Gpx4, 1:1,000), obtained from Cell Signaling Technology (Danvers, MA, USA). We visualized the labeling with a horseradish peroxidase-conjugated secondary antibody, using enhanced chemiluminescence detection (Amersham Pharmacia Biotech, Piscataway, NJ, USA). We scanned the films with an imaging system (Alliance 4.2; UVItec, Cambridge, UK), after which we used densitometry to perform a quantitative analysis of the antibodies employed, normalizing the bands to β-actin expression.

### Light microscopy

We stained 4-µm paraffin sections with periodic acid Schiff–diastase. Tubular necrosis was quantified by a pathologist who was blinded to the groups. Tubular injury, characterized by loss of the renal brush border and cast formation, as well as tubular necrosis and dilation, was graded on the basis of the proportion of the corticomedullary region involved in 10 randomly selected, non-overlapping high-power fields (× 100 magnification), on a five-point scale^19^, as follows: 0 = none; 1 = 0–10%; 2 = 10–25%; 3 = 25–50%; 4 = 50–75%; and 5 = 75–100%. For each animal, the total value is expressed as the tubular injury score.

#### Immunohistochemistry

Histological sections of renal tissue were incubated for 1 h at room temperature with the following antibodies: anti-macrophage cell-surface protein 2 (anti-Mac-2, 1:300; Cerdalene Labs, Burlington, CA, USA); anti-Klotho (1:300; ABCAM); anti-proliferating cell nuclear antigen (anti-PCNA, 1:1000; ABCAM); and anti-adiponectin (1:300; ABCAM). The reaction product was detected with a horseradish peroxidase-conjugated system (anti-rabbit polymer; Dako, Glostrup, Denmark), and the color reaction was developed with 3,3-diaminobenzidine (Sigma-Aldrich). The histological sections for PCNA and Mac-2 were divided into 25 fields (0.087 mm^2^ each; magnification, × 400), in which cells were counted, and the counts are expressed as cells/0.087mm^2^. Expression of adiponectin and Klotho was quantified by using a point grid overlaid onto the microscopy image. The result is expressed as the proportion of positively immunostained cells.

### Statistical analysis

Differences among the means of multiple parameters were analyzed by one-way analysis of variance, followed by the Kruskal–Wallis test. Comparisons between groups were analyzed by the Mann–Whitney test or Student’s *t*-test, as appropriate. Quantitative data are expressed as mean ± SEM, and values of *P* < 0.05 were considered statistically significant. Survival curves were compared by using a log-rank test. The statistical software used was GraphPad Prism, version 8.0 (GraphPad Software, Inc., San Diego, CA).

## Results

### Survival curve

**Figure 1:**
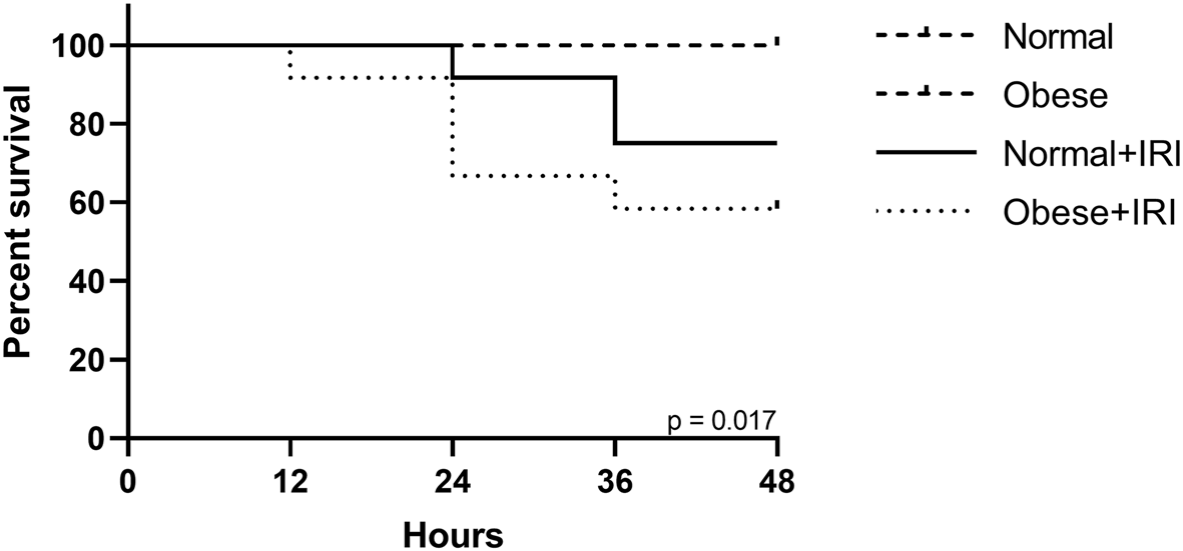
Effects of obesity on survival after ischemia–reperfusion injury (IRI)-induced acute kidney injury (AKI). Survival curves were compared by using chi-square tests.

Mortality was significantly higher in the obese + IRI group than in the normal + IRI and control groups. As illustrated in Fig. 1, five (41.7%) of the 12 mice in the obese + IRI group died, compared with three (25.0%) of the 12 in the normal + IRI group. There were no deaths in the non-IRI groups.

### Body weight

At baseline, body weights did not differ significantly among the groups. As expected, body weights at week 4 were higher among the mice that were fed a high-fat diet. After eight weeks, body weights differed significantly between the mice fed a standard diet (in the normal and normal + IRI groups) and those fed a high-fat diet (in the obese and obese + IRI groups). That difference was still significant at 48 h after the surgical procedures (Table 1). There were no statistical differences among the groups in terms of the serum levels of cholesterol.

**Table 1:** Body weight and biochemical parameters.

Parameter	Normal	Obese	Normal + IRI	Obese + IRI
	(n = 6)	(n = 6)	(n = 12)	(n = 12)
BW at baseline	22.0 ± 1.2	22.0 ± 1.0	22.8 ± 0.9	22.7 ± 0.4
BW at week 4	25.7 ± 2.1	28.6 ± 2.1	26.0 ± 0.7	32.0 ± 0.6^a^
Pre-procedure BW (g)*	28.6 ± 1.3	34.4 ± 2.0^b^	28.7 ± 0.9^c^	36.0 ± 0.5^a^
Post-procedure BW (g)^†^	27.2 ± 1.4	33.3 ± 1.7^d^	25.8 ± 9.1^e^	32.0 ± 0.7^b,f^
Serum cholesterol (mg/dL)	163 ± 26	166 ± 26	190 ± 18	196 ± 21
CrCl (mL/min/100 g BW)	0.40 ± 0.08	0.34 ± 0.06	0.20 ± 0.07	0.20 ± 0.05
Urine osmolality (mOsm/kg)	1,669 ± 625	2,648 ± 173	1,557 ± 144	1,084 ± 156^b,g^
uNGAL/urinary Cr (µg/µg)	5.7 × 10^−4^ ± 3.9 × 10^−4^	1.1 × 10^−3^ ± 7.6 × 10^−4^	0.09 ± 0.09^c,d^	0.92 ± 0.74^c,d,h^
uTBARS (nmol/mL)	530 ± 113	995 ± 196	723 ± 92	1,145 ± 158^b^

*IRI* ischemia–reperfusion injury, *BW* body weight, *CrCl* creatinine clearance, *Cr* creatinine, *uNGAL* urinary neutrophil gelatinase-associated lipocalin, *uTBARS* urinary thiobarbituric acid-reactive substances.

*After eight weeks of a standard or high-fat diet but before renal IRI or sham surgery.

^†^48 h after IRI or sham surgery.

^a^*P* < 0.0001 vs. normal and normal + IRI.

^b^*P* < 0.05 vs. Normal.

^c^*P* < 0.01 vs. Obese.

^d^*P* < 0.01 vs. Normal.

^e^*P* < 0.0001 vs. Obese.

^f^*P* < 0.0001 vs. normal + IRI.

^g^*P* < 0.05 vs. Obese.

^h^*P* < 0.01 vs. normal + IRI.

### Renal function, biochemical analysis, and histological analysis

**Figure 2:**
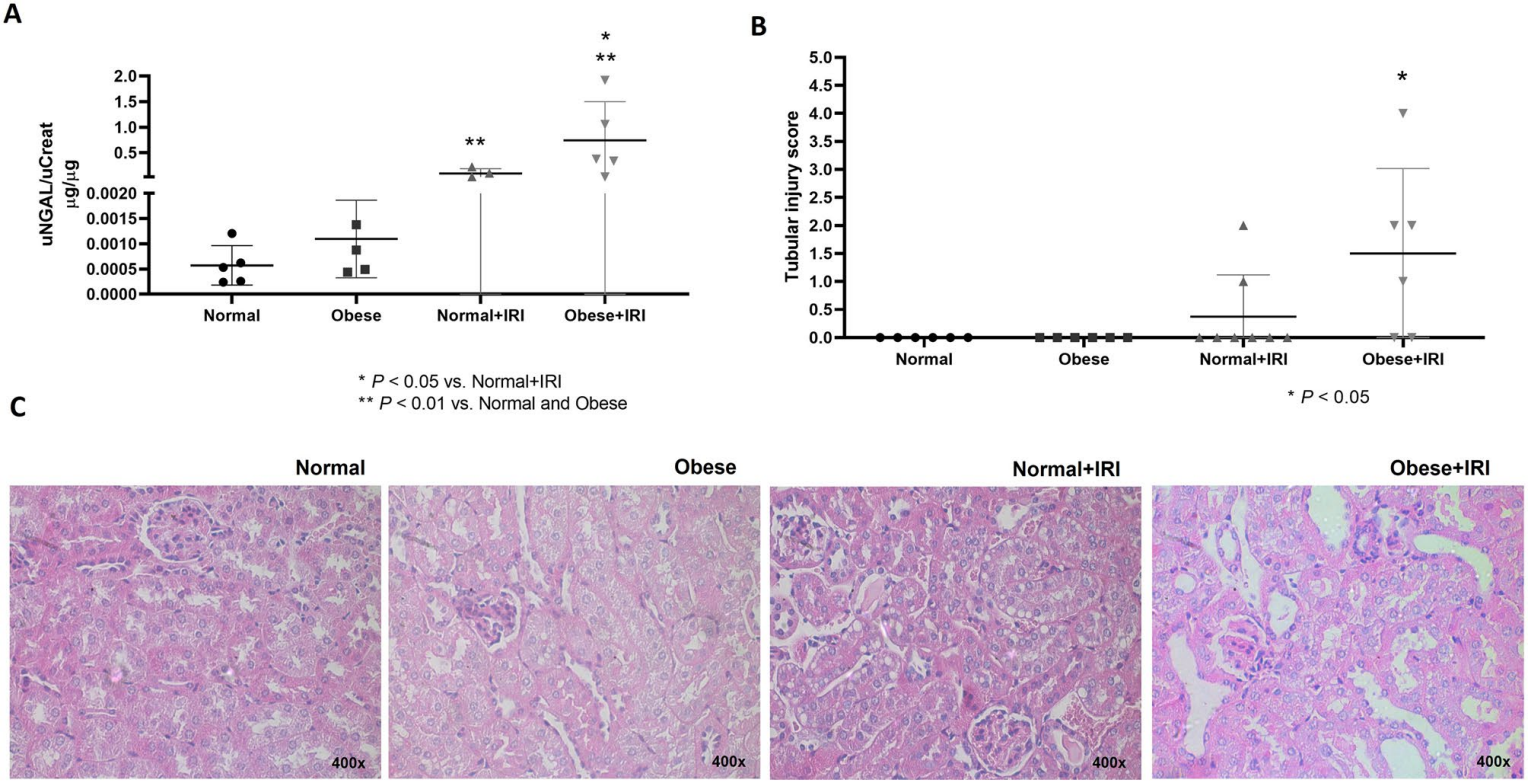
Biochemical and histological analysis at 48 h after ischemia–reperfusion injury (IRI) or sham surgery. Urinary neutrophil gelatinase-associated lipocalin (NGAL, **A**); Tubular injury score and kidney tissue sections stained with periodic acid-Schiff, by group (**B**,**C**, respectively). Differences were assessed by one-way analysis of variance, followed by Tukey’s multiple comparisons test for NGAL and the tubular injury score.

At baseline and at week 4, there were no statistical differences among the groups in terms of urinary osmolality or in terms of the urinary excretion of sodium or potassium (data not shown). At 48 h after the surgical procedures, creatinine clearance (used as a proxy for the glomerular filtration rate) did not differ significantly among the groups (Table 1). However, urinary osmolality was significantly lower in obese + IRI than in the normal and obese groups. Urinary NGAL expression were significantly higher in the normal + IRI and obese + IRI groups than in the corresponding control groups, as well as being significantly higher in the obese + IRI group than in the normal + IRI group (Fig. 2A).

**Figure 3:**
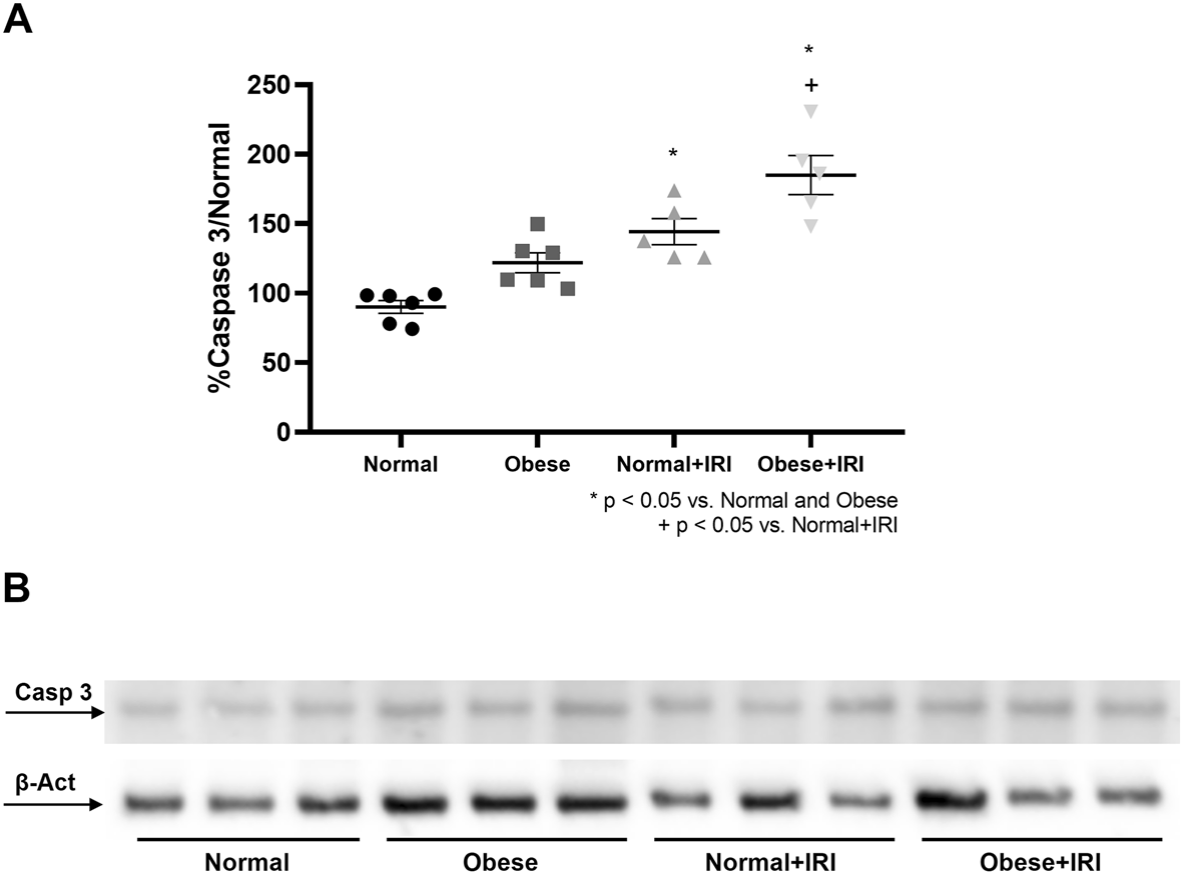
Densitometric analysis and immunoblotting for caspase 3 (**A**,**B**, respectively). Differences were assessed by one-way analysis of variance, followed by Tukey’s multiple comparisons test.

Figure 2B,C show that the extent of renal tubular damage (expressed as the tubular necrosis score) was greater in the obese + IRI group than in the normal, obese, and normal + IRI groups (1.5 ± 0.62 vs. 0.00 ± 0.00, 0.00 ± 0.00, and 0.37 ± 0.26, respectively; *P* < 0.05 for all). Semiquantitative immunoblotting (Fig. 3A,B) revealed that renal expression of caspase 3 was significantly higher in the normal + IRI and obese + IRI group mice than in the normal and obese group mice (144.2 ± 9.4 and 185 ± 14.0% vs. 90.2 ± 11.1 and 122 ± 7.2%, respectively; *P* < 0.01). That expression was also significantly higher in the obese + IRI group than in the normal + IRI group (*P* < 0.05).

### Obesity and the inflammatory response in AKI

**Figure 4:**
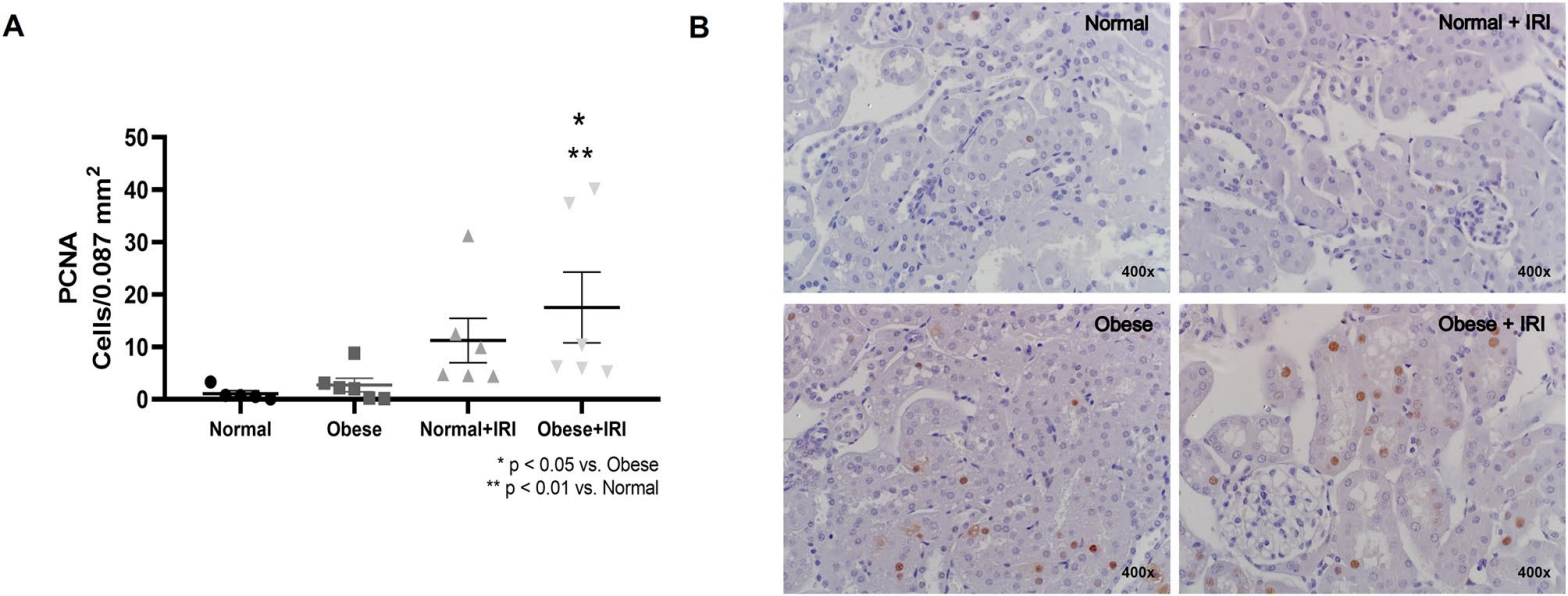
Immunohistochemical analysis of proliferating cell nuclear antigen (PCNA)-positive cell expression in mouse kidney tissue. Scatter dot plot and immunostaining (**A**,**B**, respectively). Magnification, × 400. Differences were assessed by one-way analysis of variance, followed by Tukey’s multiple comparisons test.

The number of PCNA-positive cells was significantly greater in the obese + IRI group than in the normal and obese groups (17.5 ± 6.7 cells/0.087 mm^2^ vs. 1.1 ± 0.6 and 2.7 ± 1.3 cells/0.087 mm^2^, respectively; *P* < 0.05). The PCNA-positive cell count in the normal + IRI group (11.2 ± 4.2 cells/0.087 mm^2^) did not differ significantly from those obtained for the other groups (Fig. 4A,B).

**Figure 5:**
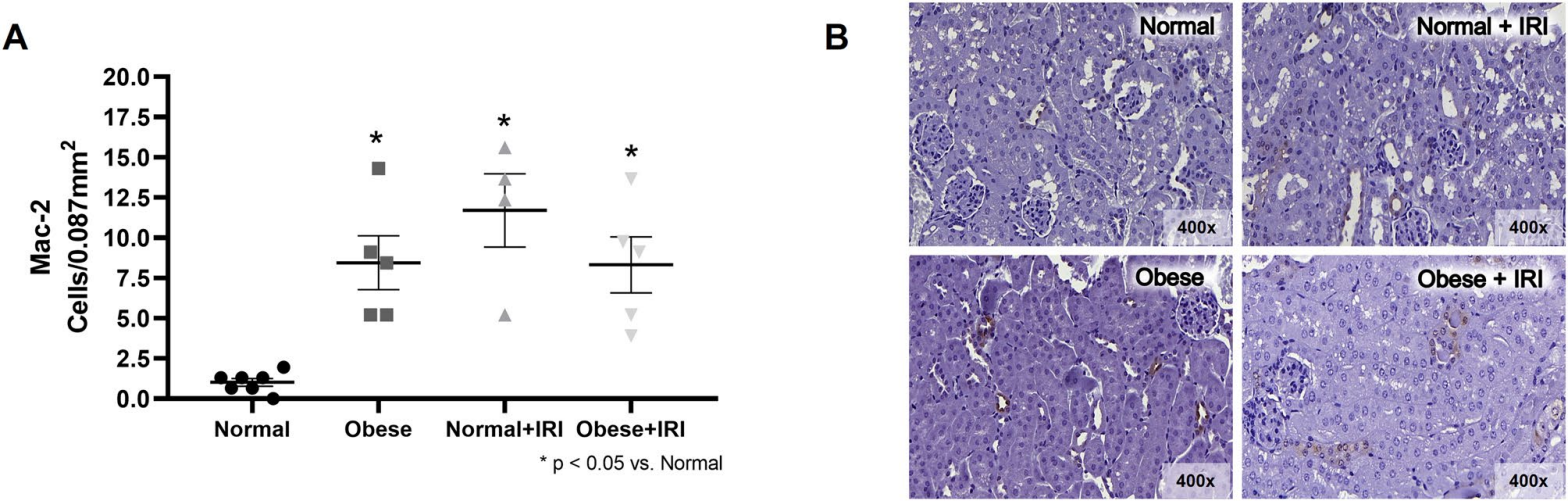
Immunohistochemical analysis of macrophage cell-surface protein 2 (Mac-2)-positive cell expression in mouse kidney tissue. Scatter dot plot and immunostaining (**A**,**B**, respectively). Magnification, × 400. Differences were assessed by one-way analysis of variance, followed by Tukey’s multiple comparisons test.

It is noteworthy that, among the mice that were not induced to AKI, macrophage infiltration into the renal interstitium, expressed as the mean number of Mac-2 + cells, was higher in the obese group than in the normal group (8.4 ± 1.7 vs. 1.0 ± 0.24 cells/0.087 mm^2^; *P* < 0.05). As expected, the mean Mac-2 + cell counts in the normal + IRI and obese + IRI groups (11.7 ± 2.27 and 8.3 ± 1.74 cells/0.087 mm^2^, respectively) were higher than that observed for the normal group (*P* < 0.05; Fig. 5A,B).

**Figure 6:**
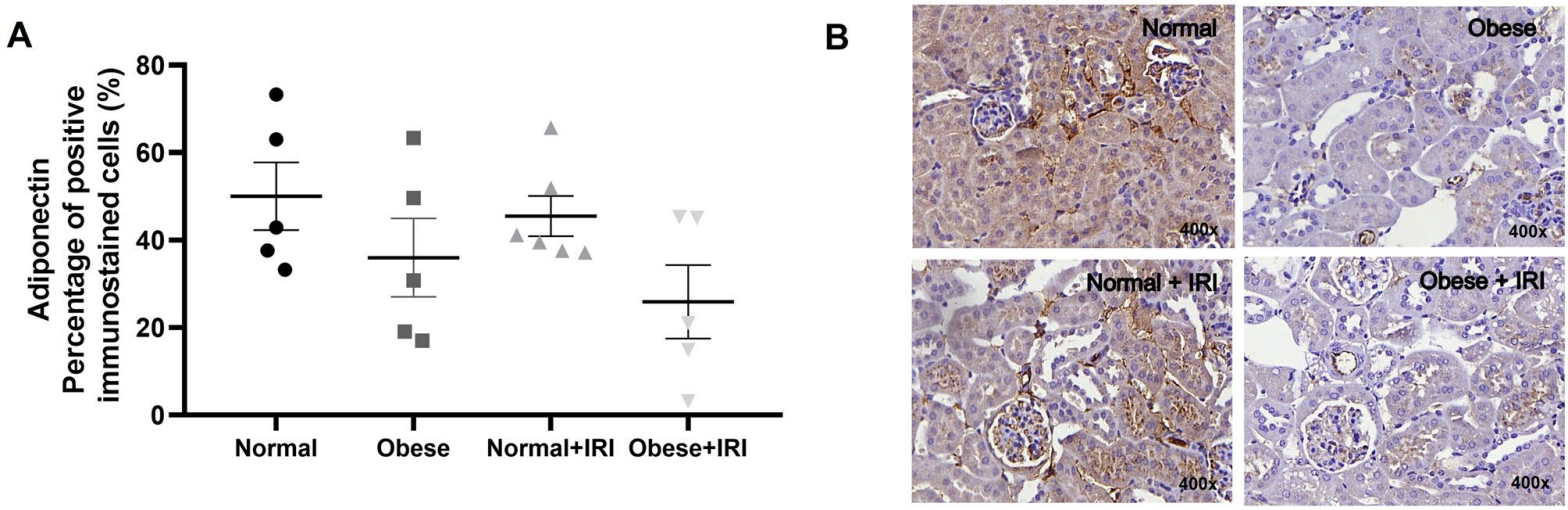
Immunohistochemical analysis of adiponectin positivity in mouse kidney tissue. Scatter dot plot and immunostaining (**A**,**B**, respectively). Magnification, × 400. Differences were assessed by one-way analysis of variance, followed by Tukey’s multiple comparisons test.

Renal expression of adiponectin (the proportion of positively immunostained cells) was lower in the obese and obese + IRI groups than in the normal and normal + IRI groups (36.0 ± 8.9 and 26.0 ± 8.4% vs. 50.0 ± 7.7 and 45.5 ± 4.6%, respectively; (Fig. 6A,B). However, the difference was not significant.

### Obesity and oxidative stress in AKI

**Figure 7:**
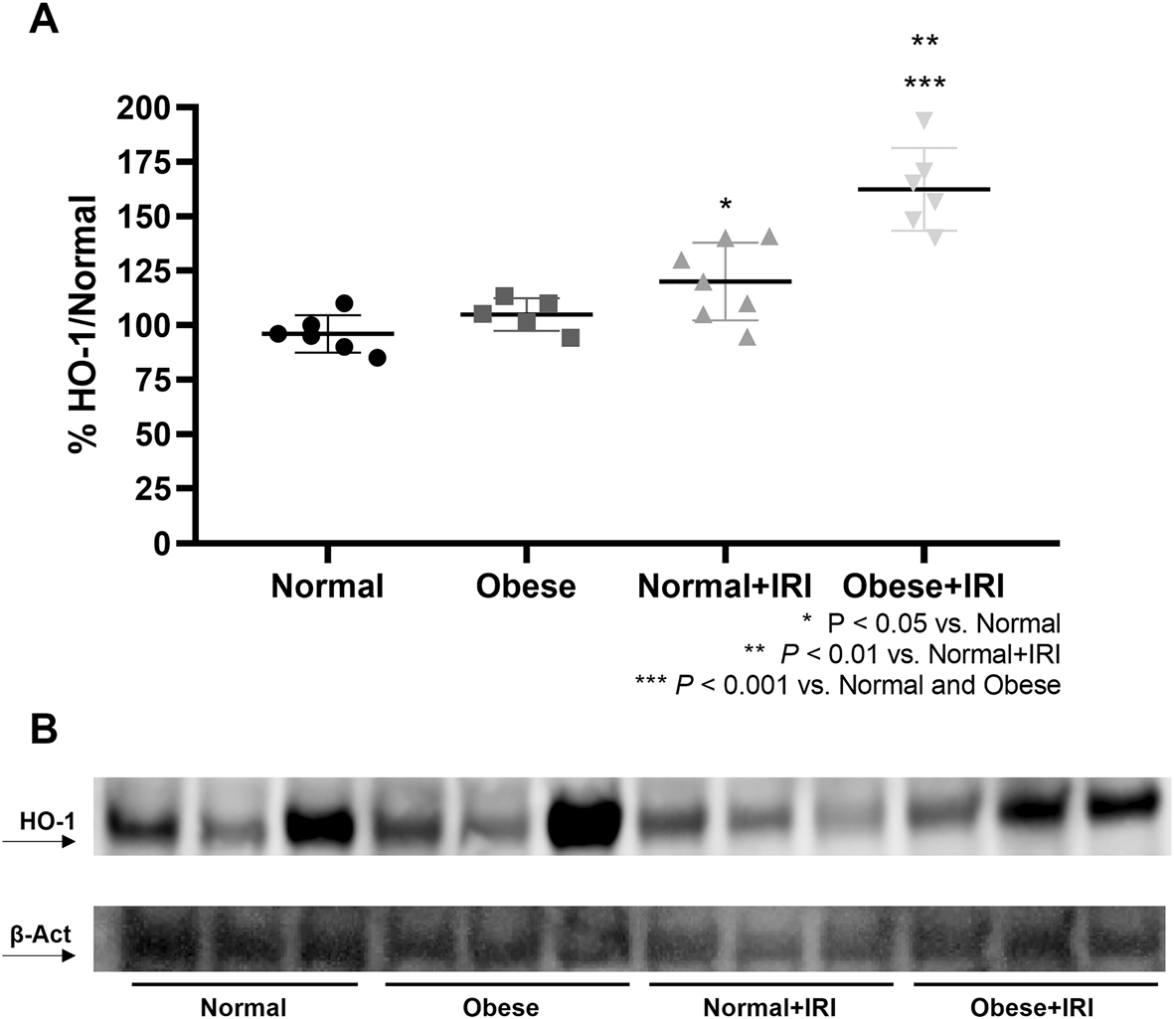
Densitometric analysis and immunoblotting for heme oxygenase-1 (HO-1; **A**,**B**, respectively). Differences were assessed by one-way analysis of variance, followed by Tukey’s multiple comparisons test.

**Figure 8:**
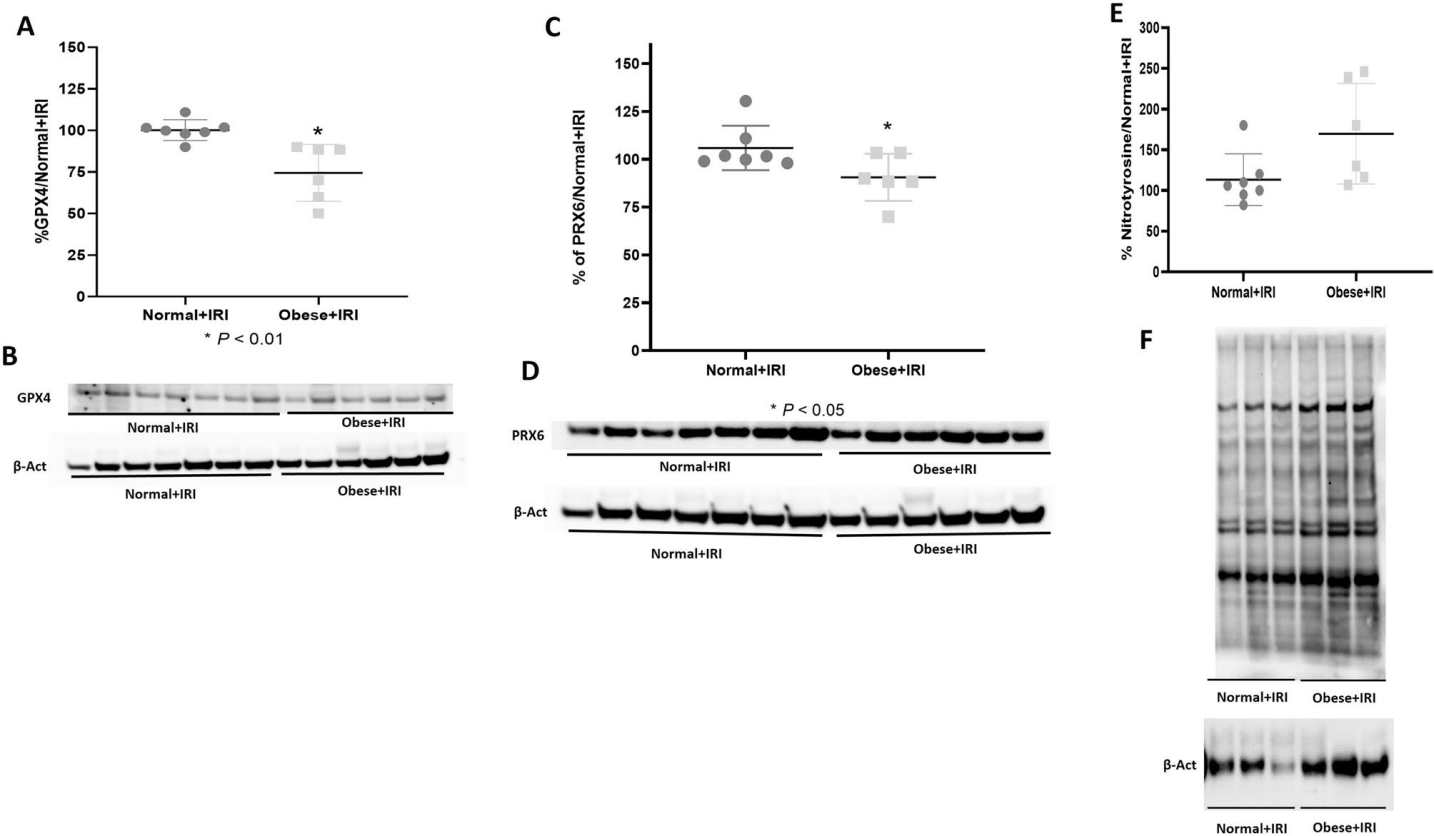
Densitometric analysis and immunoblotting for glutathione peroxidase 4 (Gpx4; **A**,**B**, respectively), peroxiredoxin 6 (Prdx6; **C**,**D**, respectively), and nitrotyrosine (**E**,**F**, respectively). Unpaired t-tests were used in all analyses.

Urinary levels of thiobarbituric acid-reactive substances (TBARS) were higher in the obese + IRI group than in the normal group (Table 1). The mean renal expression of HO-1 was higher in the obese + IRI group than in the normal and obese groups (162 ± 19% vs. 96 ± 8.6 and 105 ± 7.4%, respectively; *P* < 0.001), as well as being higher in the obese + IRI group than in the normal + IRI group (120 ± 17.8%; *P* < 0.01) and higher in the normal + IRI group than in the normal group (*P* < 0.05; Fig. 7A,B). We also evaluated lipid peroxidation, a hallmark of AKI and ferroptosis. As can be seen in Fig. 8A,B, the mean Gpx4 expression was lower in the obese + IRI group than in the normal + IRI group (74.5 ± 7.0% vs. 100 ± 2.3%; *P* < 0.01). The mean expression of Prdx6 was also lower in the obese + IRI group than in the normal + IRI group (90.6 ± 5.0% vs. 100.6 ± 4.4%; *P* < 0.05), as illustrated in Fig. 8C,D. In addition, there was a trend toward greater expression of nitrotyrosine, a marker of protein nitration, in the obese + IRI mice than in the normal + IRI mice (170 ± 25% vs. 113 ± 12%; NS), as depicted in Fig. 8E,F.

### Obesity and renal Klotho expression in AKI

**Figure 9:**
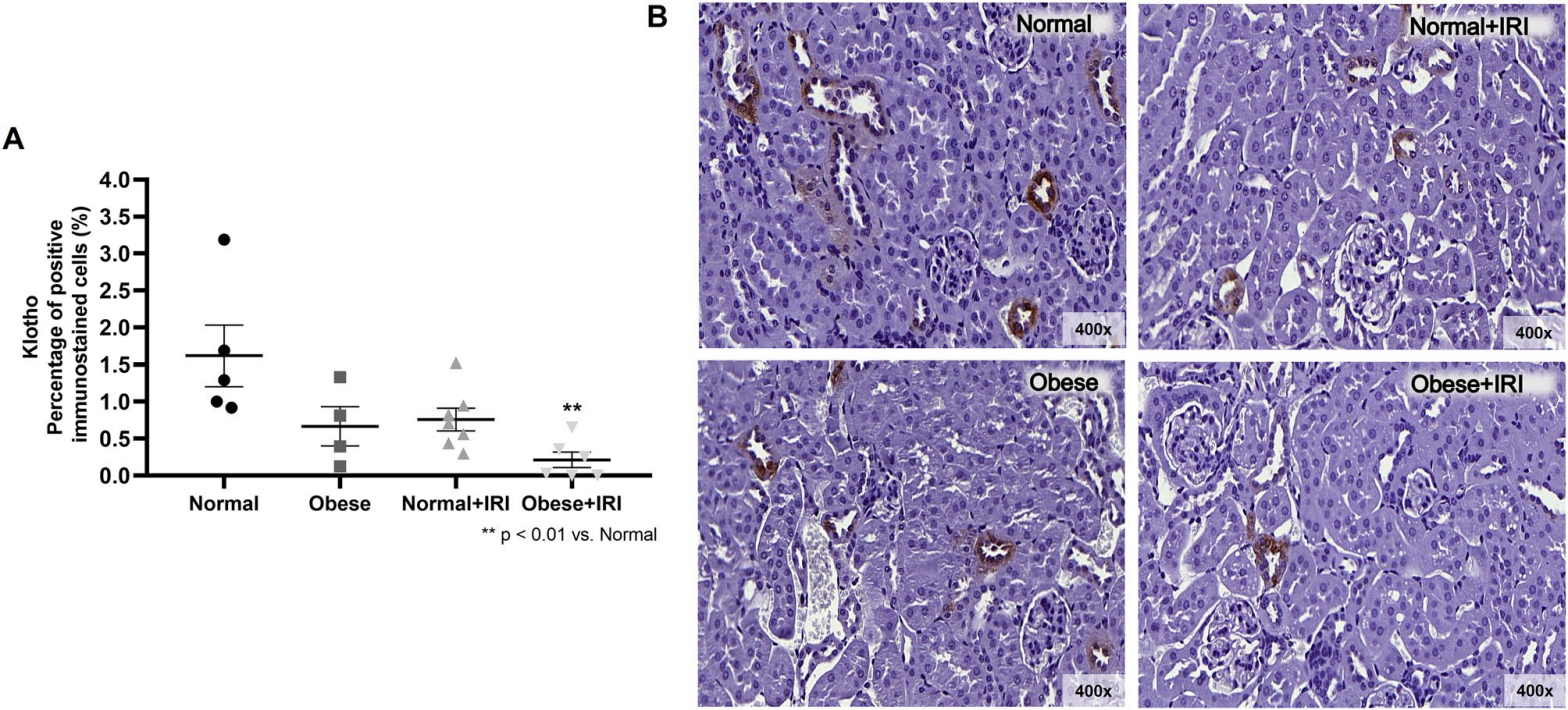
Immunohistochemical analysis of Klotho positivity in rat kidney tissue. Scatter dot plot and immunostaining (**A**,**B**, respectively). Magnification, × 400. Differences were assessed by one-way analysis of variance, followed by Tukey’s multiple comparisons test.

We found that that the mean proportion of cells showing positive immunostaining for Klotho was lower in the normal + IRI group than in the normal group (0.76 ± 0.15 vs. 1.62 ± 0.41%; *P* < 0.05). However, that difference was not significant in the comparison among all of the groups. Renal Klotho protein expression was also lower in the obese group than in the normal group (0.67 ± 0.26 vs. 1.62 ± 0.41%), although the difference was not significant in any of the analyses. The mean proportion of cells showing positive immunostaining for Klotho was lowest (0.21 ± 0.10%) in the obese + IRI group, the difference between that group and the normal and normal + IRI groups being significant (*P* < 0.05; Fig. 9A,B).

## Discussion

Here, we have demonstrated that obesity increased mortality in a mouse model of renal IRI. In addition, we found that there were no differences among the groups in terms of the glomerular filtration rate (creatinine clearance) at 48 h after IRI or sham surgery. However, obese + IRI group mice presented the lowest urinary osmolality, the highest urinary NGAL expression, the greatest renal tubular damage, and the highest caspase 3 expression. It is possible that obesity exerts those effects by upregulating oxidative stress molecules, as evidenced by the higher urinary TBARS levels and higher renal expression of HO-1 in our obese mice. In addition, lipid peroxidation, a mechanism of ferroptosis, is a critical ROS pathway, inducing tissue injury in IRI-induced AKI. We demonstrated that, among mice submitted to IRI, the expression of key inhibitors of ROS-mediated phospholipid peroxidation (Gpx4 and Prdx6) was lower in those that were obese. It has long been known that renal IRI creates a state of decreased renal Klotho protein expression^20,21^. Accordingly, we observed markedly low levels of renal Klotho protein expression in our obese mice.

Obesity is recognized as a global epidemic and as a public health crisis in various countries^21^, having also been shown to be an independent risk factor for the development of chronic kidney disease^12,23^. Although we found no differences among our groups of mice in terms of creatinine clearance, it is known that the determination of serum creatinine levels or even creatinine clearance is not the best means of estimating the glomerular filtration rate. There have been studies showing that tubular secretion of creatinine is higher among obese patients than among non-obese patients^24^. However, to our knowledge, there have been no animal studies showing that obesity aggravates IRI-induced AKI. In the present study, we demonstrated that obesity leads to kidney damage, resulting in tubular necrosis and apoptosis, as well as greater urinary expression of NGAL. In a previous study, involving Zucker rats, the obese rats were found to develop AKI within the first 24 h after orthopedic trauma, although the same did not occur among the lean rats^25^. In critically ill patients, obesity is known to be an independent risk factor for the development of AKI. In a recent review of the literature, Schiffl^26^ discussed the survival paradox of obese critically ill patients with AKI. The author defined the evidence that obesity improves survival among critically ill patients with AKI as “a paradox within a paradox”. Because there are no clearly defined pathophysiological mechanisms, there is still controversy regarding the question of whether that survival benefit is attributable to biological reactions or to methodological errors. Obese patients often also have metabolic syndrome, diabetes, dyslipidemia, hypertension, or cardiovascular disease^27,28^. In our mouse model of IRI-induced AKI, the animals did not develop diabetes or dyslipidemia. Therefore, we can speculate that the obesity per se might be an aggravating factor in IRI-induced AKI. Despite not having been submitted to IRI, the obese group mice showed higher Mac-2 inflammatory cell counts in renal tissue. Adipocytes store excess energy by undergoing hypertrophy. Visceral adipocytes undergo hypertrophy when storing additional lipids, becoming poorly vascularized and hypoxic, resulting in increased inflammatory cytokine production, immune cell infiltration, as well as cell stress and apoptosis^30^. When that occurs, fat is stored in the kidneys, liver, pancreas, heart, skeletal muscle, and other tissues, leading to a condition known as lipotoxicity. Tissue-resident and infiltrating macrophages regulate the innate immune system, which plays a crucial role in inflammation in adipose tissue. It has been shown that macrophage activation and macrophage infiltration into adipose tissue are both more pronounced among patients who are obese^29^. Lipids are a significant component in the normal kidney, accounting for approximately 3% of its wet weight^30^. In a model of high-fat diet-induced obesity, Laurentius et al.^31^ demonstrated that the number of infiltrating monocytes/macrophages was significantly higher in the kidneys of rats fed a high-fat diet than in those of rats fed a control diet. We found it interesting that, in our study, the Mac-2 + cell counts were higher in the obese group than in the normal group, despite the fact that the mice in both of those groups underwent sham surgery (i.e., were not induced to AKI), and that the obese + IRI group counts were comparable to those observed for the obese and normal + IRI groups. In the present study, an anti-Mac-2 antibody was used in order to identify macrophages in renal tissue. In the kidney, the antibody labeled a fraction of the CSF1R + and CX3CR1 + macrophages but also stained tubular epithelial cells^32^, which might have interfered with the identification of macrophage infiltration.

Serum levels of the protein adiponectin are lower in obese individuals, especially in those with visceral fat accumulation and altered lipid metabolism, which is paradoxical because adiponectin is derived from adipocytes^33^. In another mouse model of acute IRI, Tsugawa-Shimizu et al.^34^ detected adiponectin in the endothelium of the renal arterioles and in the peritubular spaces of the renal cortex, as well as in the inner and outer renal medulla. The authors found that there was more renal tubular damage and greater vascular permeability in adiponectin-knockout mice than in wild-type mice. They suggested that adiponectin, by binding to T-cadherin, plays a major role in preserving the capillary network and mitigating renal tubular injury^34^. In the present study, renal expression of adiponectin was lower in the obese and obese + IRI groups than in the other groups, although the difference was not statistically significant. We believe that the lack of statistical difference was attributable to the great variability within the groups. The phospholipid hydroperoxide Gpx4 plays a crucial role in preventing lipid peroxidation in cell membranes, and its inactivation promotes ferroptosis and ROS production^35^. Here, we have demonstrated that Gpx4 expression was found to be lower in the mice with AKI that were obese than in those that were not. The peroxiredoxin Prdx6, which regulates redox and is crucial for cell homeostasis, is multifunctional, with the ability to neutralize peroxides and stimulate the production of ROS^36^. Overexpression of Prdx6 has been shown to suppress ferroptosis^37^. In the present study, Prdx6 expression was found to be lower in the mice with AKI that were obese than in those that were not. Ferroptosis and lipid peroxidation might be involved in the worsening of AKI seen in the obese mice evaluated in the present study. Among those mice, the protein expression of nitrotyrosine, which is produced by tyrosine nitration induced by nitrogen dioxide, peroxynitrite anion, or other ROS^38^, tended to be higher than among the non-obese mice.

In a very elegant study, Cui et al.^4^ analyzed the association between the visceral adiposity index and serum levels of the anti-aging protein Klotho protein, using (United States) National Health and Nutrition Examination Survey data. The authors found that, among adults in the United States, there was a non-linear association, as well as a dose–response relationship, between serum Klotho levels and the visceral adiposity index. They showed that a there was a negative correlation between the two factors, albeit only when the visceral adiposity index below 3.21. Among the individuals with a visceral adiposity index between 0.29 and 3.21, serum Klotho levels decreased as visceral adiposity increased, and those individuals were more prone to aging-related syndromes. In the present study, the mice that were fed a high-fat diet showed lower renal Klotho expression than did those that were fed a standard diet, although the difference was not statistically significant. In an in vitro study, Xiong et al.^39^ demonstrated that D-galactose induces aging in H9C2 cells. The authors found that, in such cells, treatment with D-galactose increased β-galactosidase activity, reduced cell viability, increased oxidative stress, reduced the numbers of mitochondrial cristae, and decreased Gpx4 expression. They also demonstrated that subsequent treatment with Klotho abolished D-galactose-induced aging in H9C2 cells, likely because of its ability to increase expression of ferroptosis-associated proteins like GPx4.

Hu et al.^20^ showed that IRI reduces Klotho in the kidneys, urine, and blood of rodents. The mice in our normal + IRI group also presented lower renal Klotho protein expression than did those in our normal group. Nevertheless, that expression was much lower in our obese + IRI group. Therefore, we can surmise that obesity is a state of Klotho deficiency, which becomes much more pronounced when obese animals are submitted to IRI. Klotho deficiency has been shown to increase endogenous ROS generation and accentuate oxidative stress^40,41^. Conversely, overexpression of Klotho induces resistance to oxidative stress^22^. In addition, Klotho administration has been shown to effectively reduce oxidative stress and preserve mitochondrial function in mice^41^.

## Conclusion

In obesity, increased oxidative stress and ferroptosis might be the mechanisms that aggravate IRI. In obese individuals with AKI, Klotho could be a therapeutic target.

### Supplementary Information

Supplementary Information.

## Data Availability

The datasets generated during and/or analyzed during the current study are available from the corresponding author on reasonable request.
