# Author Correction: Obesity aggravates acute kidney injury resulting from ischemia and reperfusion in mice

**DOI:** 10.1038/s41598-024-61997-1

**Published:** 2024-05-15

**Authors:** Igor Oliveira da Silva, Nicole K. de Menezes, Heloisa D. Jacobina, Antonio Carlos Parra, Felipe Lima Souza, Leticia Cardoso Castro, Joris J. T. H. Roelofs, Alessandra Tammaro, Samirah Abreu Gomes, Talita Rojas Sanches, Lucia Andrade

**Affiliations:** 1 Laboratory of Basic Science in Renal Diseases (LIM-12), Division of Nephrology, University of São Paulo School of Medicine, São Paulo, Brazil; 2 Laboratory of Cellular Genetic and Molecular Nephrology, Division of Nephrology, Av. Dr. Arnaldo, 455, 3º Andar, sala 3310, University of São Paulo School of Medicine, São Paulo, SP CEP 01246-903 Brazil; 3 Department of Pathology, Amsterdam UMC, Location AMC, University of Amsterdam, Amsterdam, the Netherlands; 4 Amsterdam Cardiovascular Sciences, University of Amsterdam, Amsterdam, The Netherlands

Correction to: *Scientific Reports* 10.1038/s41598-024-60365-3, published online 29 April 2024

The original version of this Article contained an error in the spelling of the author Joris J.T.H. Roelofs which was incorrectly given as Joris Roelofs.

Additionally, the Article omitted an affiliation for Joris J.T.H. Roelofs. The correct affiliations are listed below.

Department of Pathology, Amsterdam UMC, Location AMC, University of Amsterdam, Amsterdam, the Netherlands

Amsterdam Cardiovascular Sciences, University of Amsterdam, The Netherlands

The original Article has been corrected.

