# Supplementary figures and images for: Obesity aggravates acute kidney injury resulting from ischemia and reperfusion in mice
Source: Sci Rep. 2024 Apr 29;14:9820. doi: 10.1038/s41598-024-60365-3 (PMC11059346; doi:10.1038/s41598-024-60365-3)

HO-1

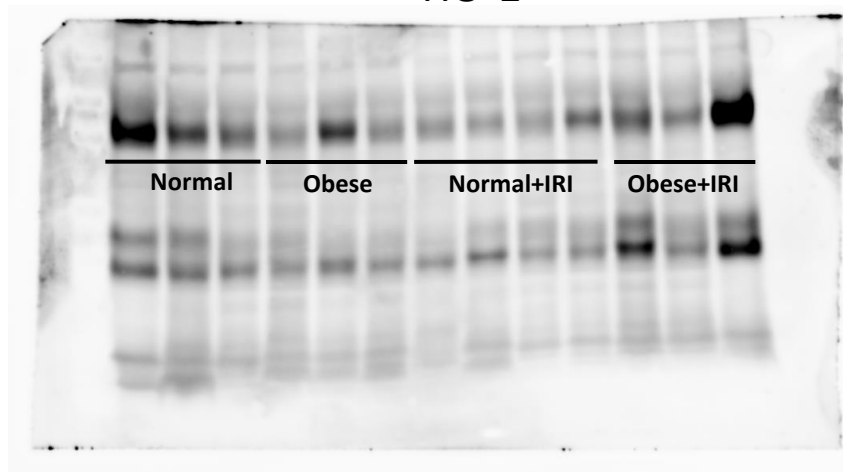

GPDH

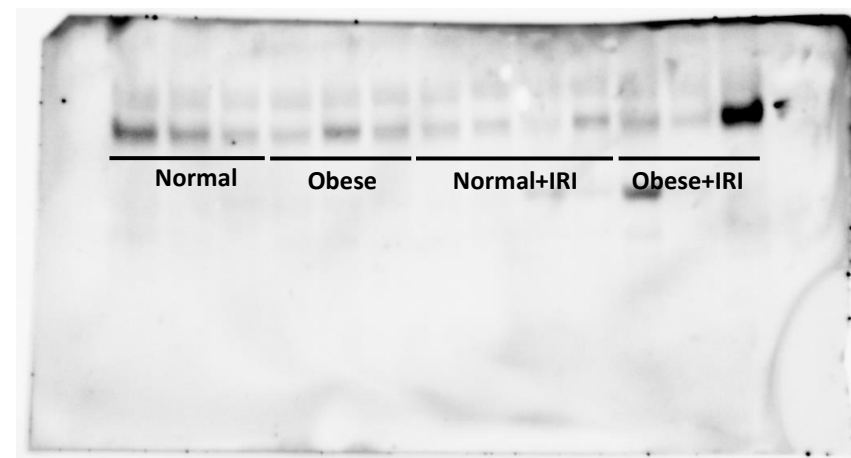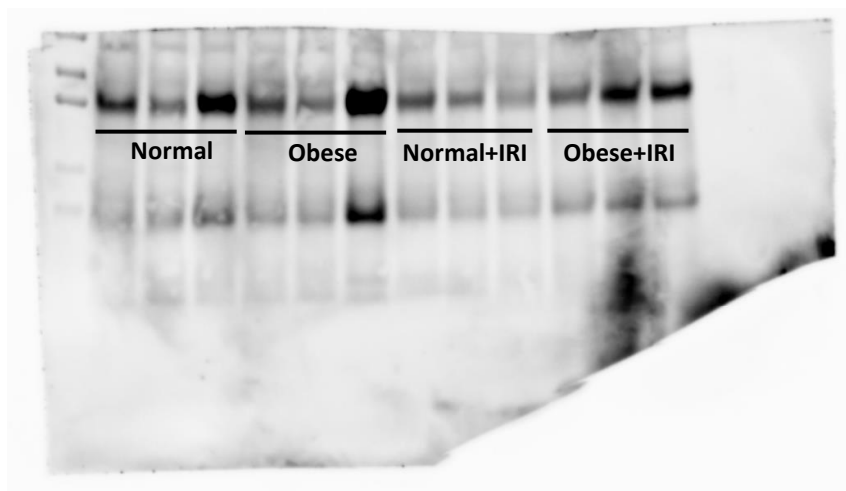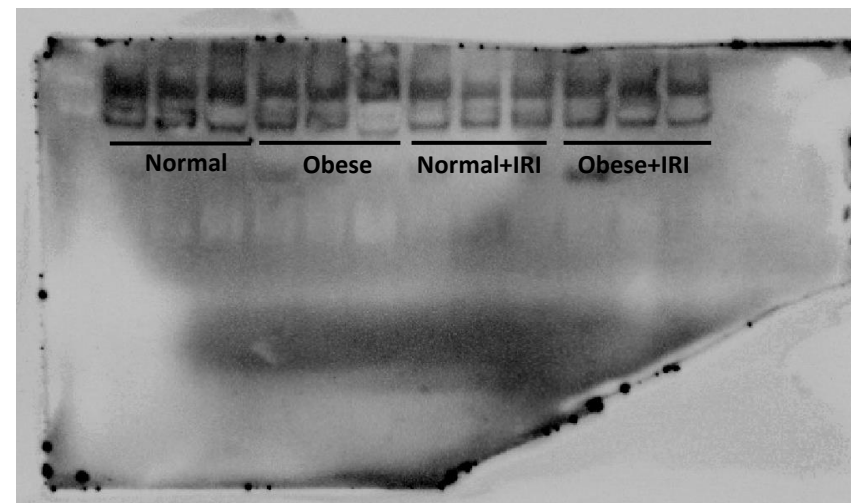

Caspase 3

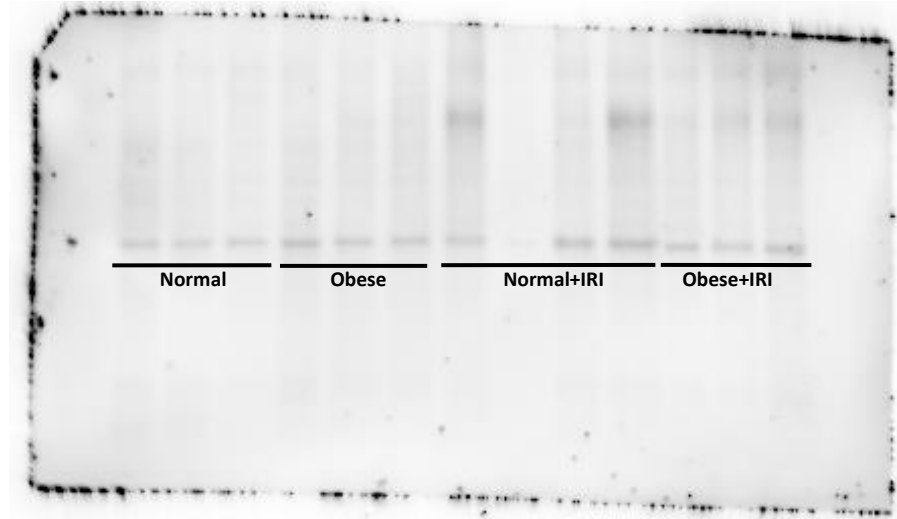

$\beta$ -Act

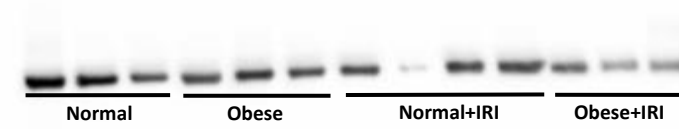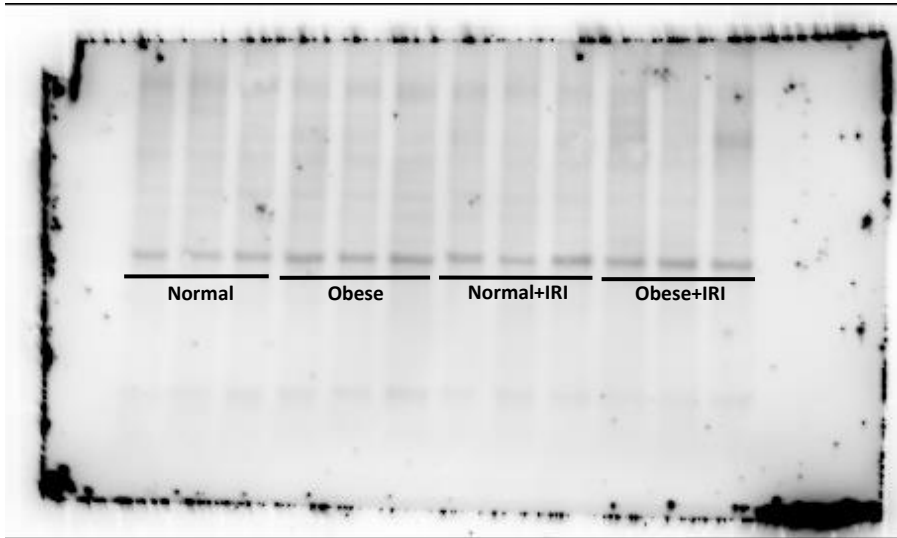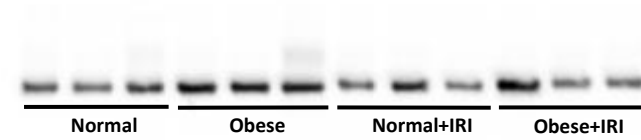

Nitrotyrosine

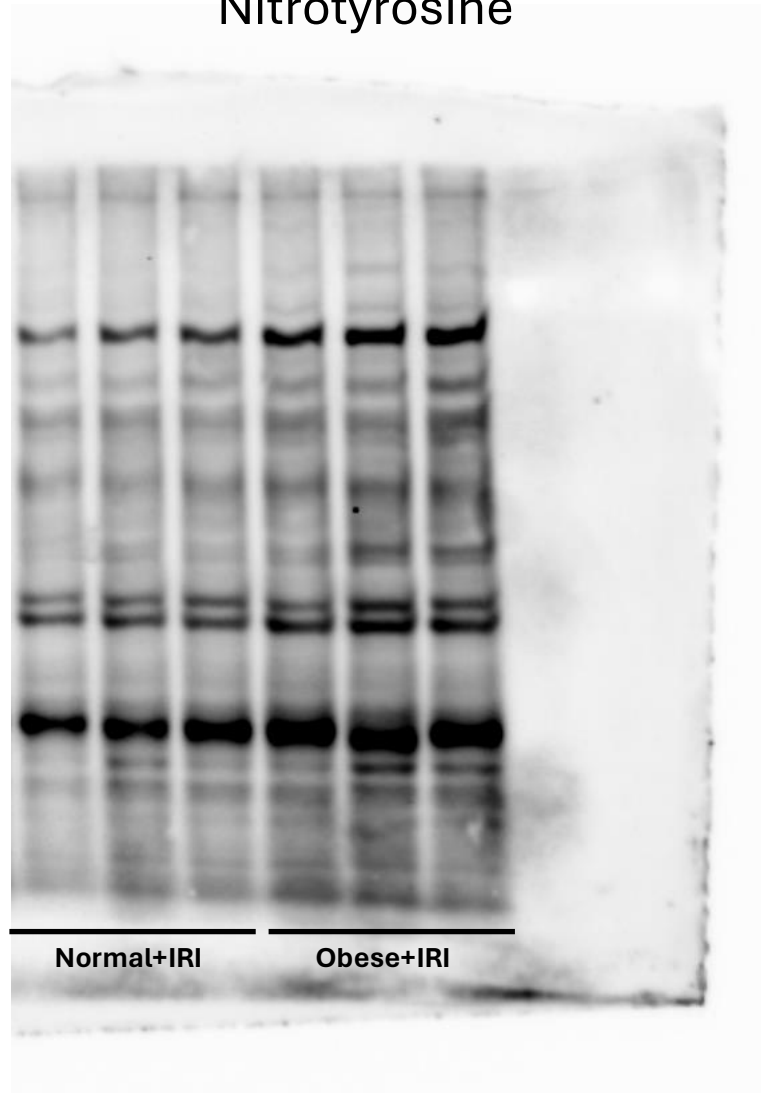

$\beta$ -Act

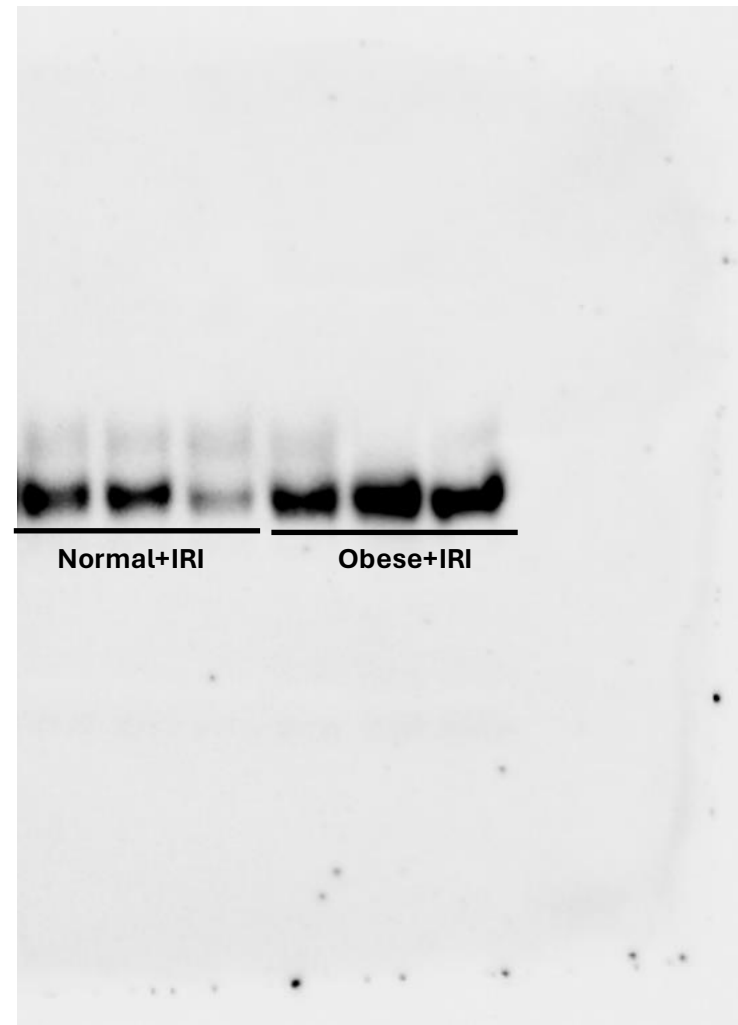

GPX4

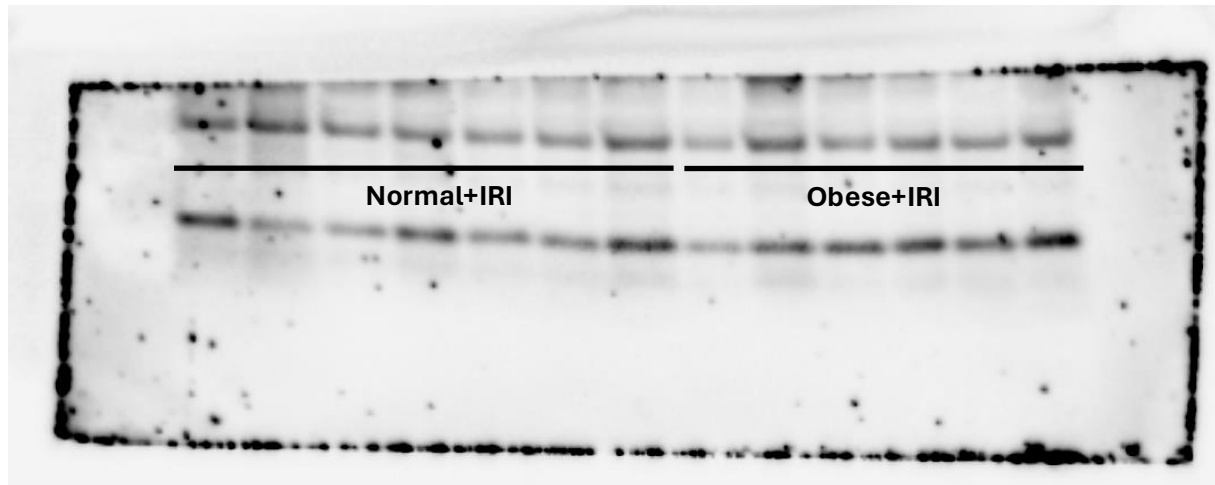

$\beta$ -Act

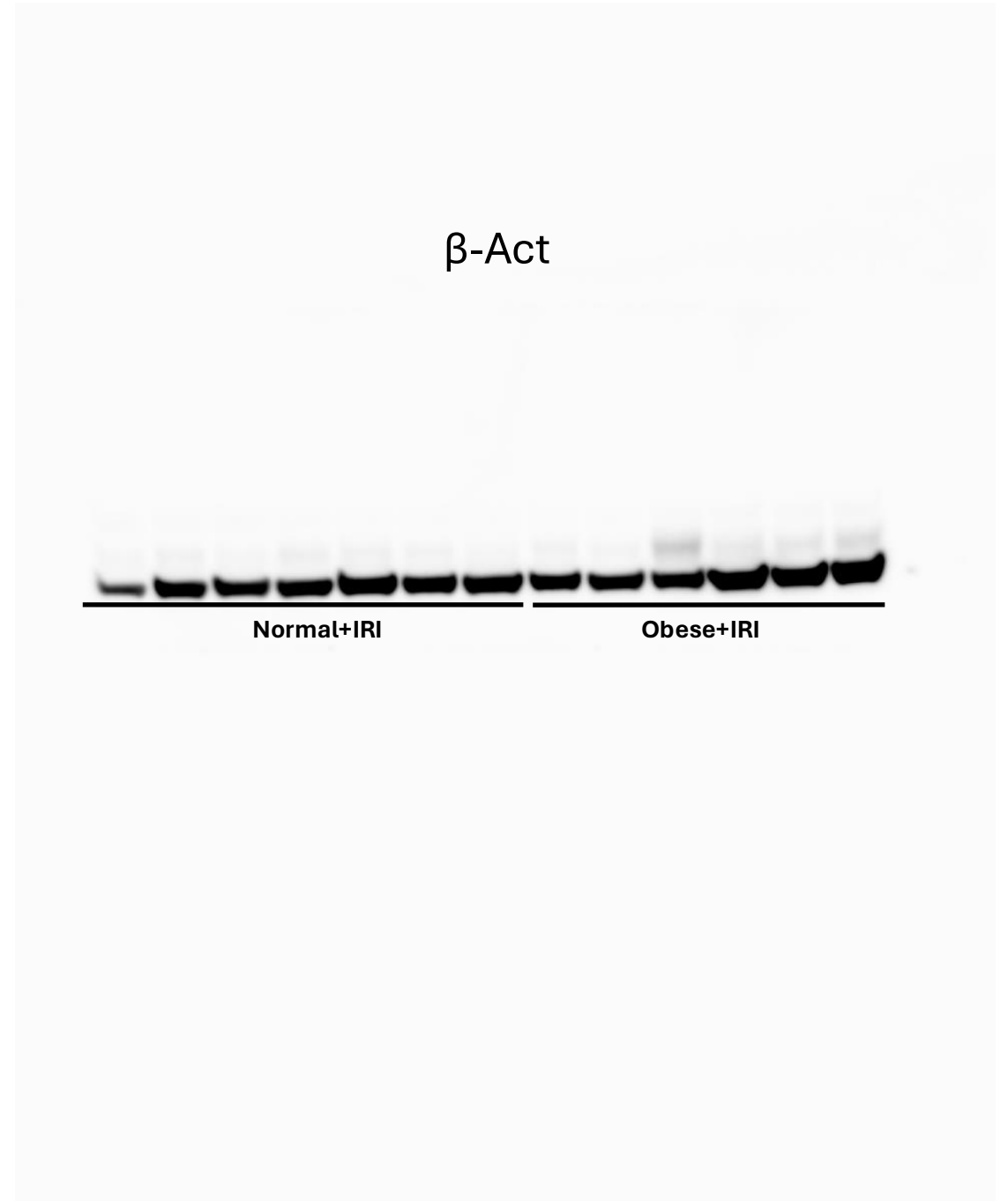

PRX6

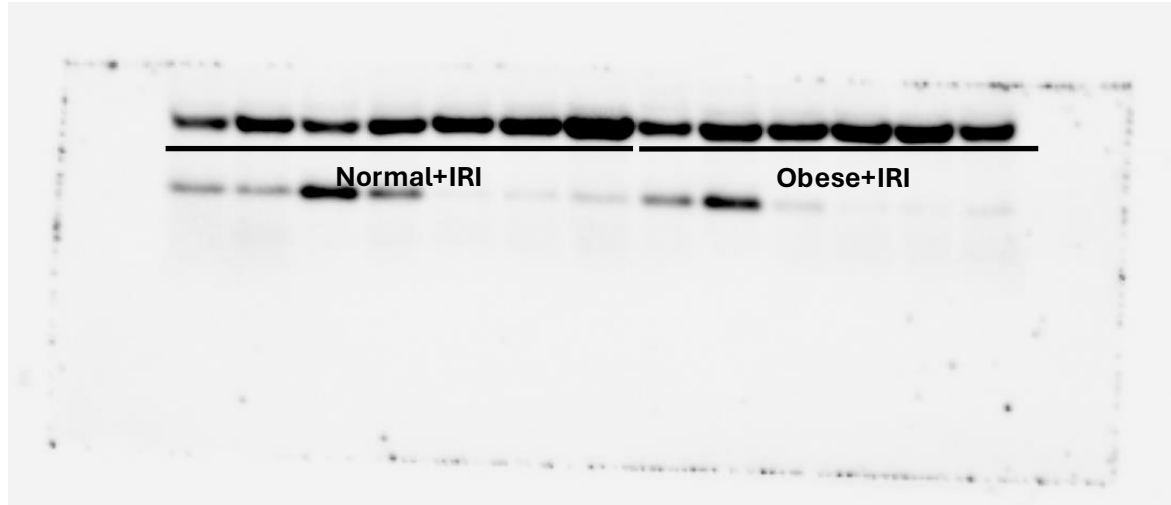

$\beta$ -Act

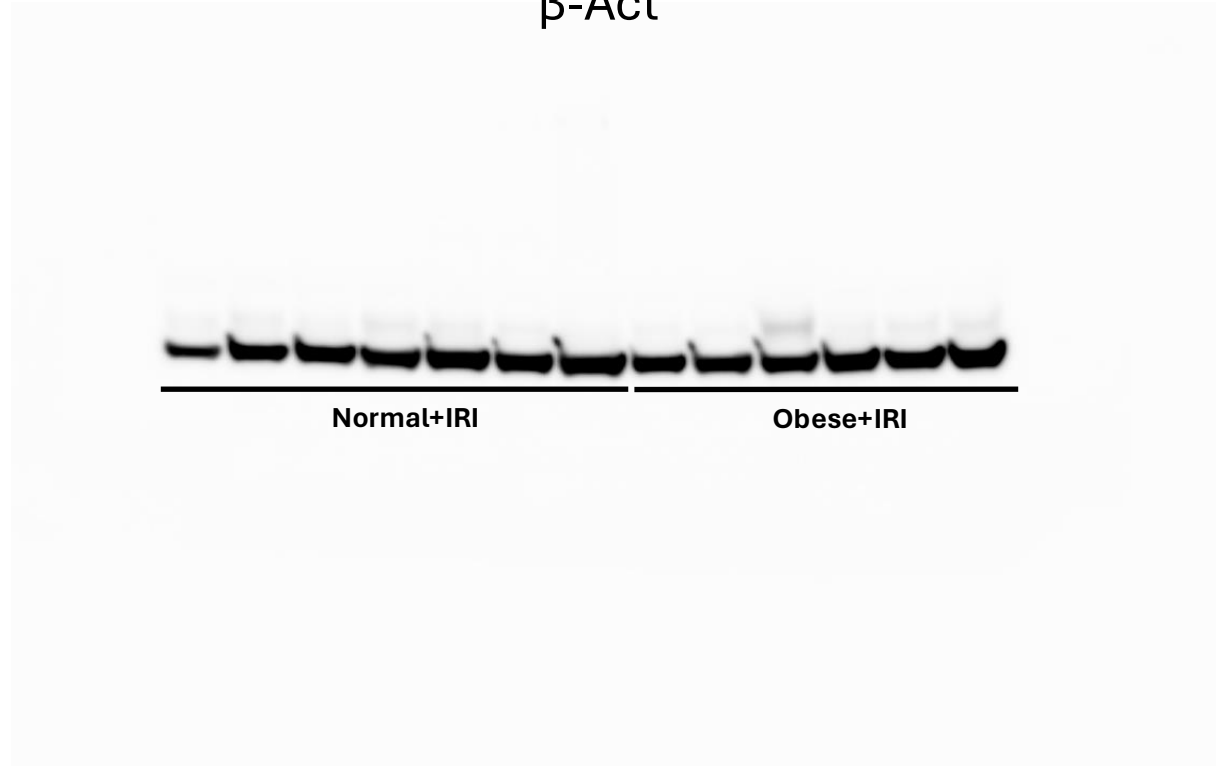

Supplement: Supplementary file 1 — Supplementary Information. [file 41598_2024_60365_MOESM1_ESM.pdf]
